# Hospital Festivals and Meetings

**Published:** 1895-05-18

**Authors:** 


### HOSPITAL FESTIVALS AND MEETINGS.

The annual meeting of the British Home for Incurables was held at the Cannon Street Hotel on May 9th, Earl Amherst in the chair. The report stated that in the past year the income had amounted to a larger sum than had ever before, in the history of the charity, been received in one year, £25,057. The greatest portion of this money had been devoted to the new home opened at Streatham last July by the Princess of Wales. Here there were still some empty rooms waiting only for funds to afford accommodation for 20 more inmates. At the end of last year the number of patients in the home numbered 47, and the pensioners 282. The report was unanimously adopted, and the opportunity was taken to present Mr. Gofton Salmond, the secretary, with a cheque for 100 guineas and an illuminated address on vellum, in consideration of his many labours for the institution during the past 14 years, and especially to commemorate the energy he had displayed in raising funds for the new building.

### PUBLIC HOSPITAL AND DISPENSARY, SHEFFIELD.

The chief hospital event of the past week has been the visit of the Duke and Duchess of York to Sheffield, to open a new wing of the Public Hospital and Dispensary, and to lay the foundation-stone of the new Nurses' Home. The old hospital, which is gradually being pulled down to make way for a building more adapted to modern uses, was established in 1832, and has done good and useful work. The new hospital, when completed, will be in the shape of the letter E, and it is the eastern block which is now finished. The streets of Sheffield were decorated and in holiday garb, and almost an universal holiday was observed on Saturday last, the Duke and Duchess of York receiving everywhere a most cordial welcome. Their Royal Highnesses inspected the wards and various departments of the new wing, being received by the Duke of Norfolk, the president of the hospital, and a gold, diamond studded key having been presented to the Duchess, Her Royal Highness unlocked the main entrance and declared the wing open. Everyone then adjourned to the site of the corner stone of the new home, where an address was presented, and prayers having been read by the Archbishop of York, and a hymn sung, the Duke of York laid the stone, and then announced amid much enthusiasm that Her Majesty the Queen had given her approval to the hospital being in future known as "The Royal Sheffield Hospital." Cheques towards the building fund were afterwards received by the Duchess of York. The accommodation provided by the present building is 105 beds, and at the completion of the new scheme these will be increased by another 60 or 70. The cost has been estimated at £50,000, of which sum £26,000 has been already collected, the Duke of Norfolk having contributed £5,000.

### COOKERY AND FOOD EXHIBITION.

The eighth Universal Cookery and Food Exhibition was opened at the Portman Rooms last week by Prince Edward of Saxe-Weimer. It is eight years ago since the first exhibition was held in Willis's Rooms, organised by the Cookery and Food Association, and much has been done in this period through the efforts of the association to spread a general knowledge of the nature and properties of food and the right way to prepare it among all classes. In London alone instruction is now being given to some thirty-three thousand children in elementary schools and in the London County Council Cookery School. These exhibitions serve a useful purpose by keeping up interest in all branches of the culinary art, and affording scope for healthy competition. At the Portman Rooms on this occasion there was much that was interesting to note on all sides. Army cooking was demonstrated by the Army School of Cookery, by permission of the General Officer commanding at Aldershot. Lectures were given on the various ways of preparing potatoes; and M. E. Pouard, of the Queen's Guard, St. James's Palace, gave demonstrations. Very interesting, too, was it to watch the school children busily and deftly preparing their special dishes for the various competitions, and most creditably they appeared to be carrying out the work. Some excellent cooking ranges were shown by Messrs. Sugg, and there was an interesting stall devoted to appliances for cooking by electricity, from Messrs. Campton and Co. A preparation specially to be noted as a novelty was "Uruguay dried beef," dried in the sun or artificially, and exhibited by Messrs. Clouzet and Duclos, of Monte Video. To cook it the meat is simmered until it swells to its original size. The price is fourpence a pound. In the first two rooms were specimens of all kinds of kitchen and other utensils, among which we noticed more than one new and useful idea, and laid out luncheon and dinner tables. Demonstrations were given in all kinds of special cookery, household and invalid, for hospitals and schools, on large and small scales, and many substantial prizes were subscribed in the various sections of the exhibition for the best dishes and arranged tables, &c. Many people visited the exhibition, and must have profited by the experience there gained. Half the proceeds are to be devoted to the advancement of the objects of the association, and the rest will go towards providing dinners for poor children, the association having already contributed nearly £1,500 to various charities on account of previous exhibitions.

